# Presence of intrinsically disordered proteins can inhibit the nucleation phase of amyloid fibril formation of Aβ(1–42) in amino acid sequence independent manner

**DOI:** 10.1038/s41598-020-69129-1

**Published:** 2020-07-23

**Authors:** Koki Ikeda, Shota Suzuki, Yoshiki Shigemitsu, Takeshi Tenno, Natsuko Goda, Atsunori Oshima, Hidekazu Hiroaki

**Affiliations:** 1 Laboratory of Structural and Molecular Pharmacology, Graduate School of Pharmaceutical Sciences, Nagoya University, Furocho, Chikusa-ku, Nagoya, Aichi 464-8601 Japan; 2 Laboratory of Structural Physiology, Graduate School of Pharmaceutical Sciences, Nagoya University, Furocho, Chikusa-ku, Nagoya, Aichi 464-8601 Japan; 3 School of Life Science and Technology, Tokyo Institute of Technology, Nagatsuda, 4259, Midori-ku, Yokohama, Kanagawa 226-8503 Japan; 4 Cellular and Structural Physiology Institute (CeSPI), Nagoya University, Furocho, Chikusa-ku, Nagoya, Aichi 464-8601 Japan; 5 BeCellBar LLC, Business Incubation Building, Nagoya University, Furocho, Chikusa-ku, Nagoya, Aichi 464-8601 Japan

**Keywords:** Biophysics, Intrinsically disordered proteins, Peptides, Proteins, Peptides, Protein folding, Proteins

## Abstract

The molecular shield effect was studied for intrinsically disordered proteins (IDPs) that do not adopt compact and stable protein folds. IDPs are found among many stress-responsive gene products and cryoprotective- and drought-protective proteins. We recently reported that some fragments of human genome-derived IDPs are cryoprotective for cellular enzymes, despite a lack of relevant amino acid sequence motifs. This sequence-independent IDP function may reflect their molecular shield effect. This study examined the inhibitory activity of IDPs against fibril formation in an amyloid beta peptide (Aβ(1–42)) model system. Four of five human genome-derived IDPs (size range 20 to 44 amino acids) showed concentration-dependent inhibition of amyloid formation (IC_50_ range between 60 and 130 μM against 20 μM Aβ(1–42)). The IC_50_ value was two orders of magnitude lower than that of polyethylene-glycol and dextran, used as neutral hydrophilic polymer controls. Nuclear magnetic resonance with ^15^ N-labeled Aβ(1–42) revealed no relevant molecular interactions between Aβ(1–42) and IDPs. The inhibitory activities were abolished by adding external amyloid-formation seeds. Therefore, IDPs seemed to act only at the amyloid nucleation phase but not at the elongation phase. These results suggest that IDPs (0.1 mM or less) have a molecular shield effect that prevents aggregation of susceptible molecules.

## Introduction

In living cells, almost all proteins must co-exist with other proteins and other biological molecules, such as nucleic acids and phospholipid vesicles, which are present at extremely high concentrations. For example, a typical cell may contain ~ 25% protein by volume, in addition to certain amounts of RNA^1,2^. These macromolecules occupy approximately 30% of the cellular volume, suggesting that steric exclusion imposed by a lack of space in solution must result in significant thermodynamic alterations in proteins. The cellular environment therefore represents a rather unusual condition quite unlike an ideal solution. Nevertheless, legacy researchers commonly set their experimental conditions for biophysical protein studies in a range between 10 μg/mL and 10 mg/mL in order to simplify the analysis of experiments by neglecting any crowding effect.

A half century ago, the concept “macromolecular crowding” was raised and then extensively studied. Many phenomena caused by macromolecular crowding are seemingly well explained by the excluded volume effect of hydrophilic macromolecules^1,3–6^. Accordingly, many basic studies have estimated the contribution of the excluded volume effect using high concentrations of neutral hydrophilic polymers, such as polyethylene glycol (PEG) and dextran (DEX), as model crowding agents. By contrast, apart from bovine serum albumin (BSA), proteins have not been widely studied as molecular crowders.

We have a specific interest in the properties of intrinsically disordered proteins (IDPs) as macromolecular crowding agents. The nomenclature IDP identifies these as another class of proteins in the post-genomic era that are exceptions to the protein “sequence–structure–function” dogma^7–11^. This is because IDPs and the intrinsically disordered regions (IDRs) within their structures do not adopt unique and compact three-dimensional structures even under physiological conditions. Despite their lack of a compact structure, IDPs are proposed to have a variety of biological functions in increasing numbers of reports. These functions include control of transcription, translation, signaling cascades, and liquid–liquid phase separations^12,13^.

The relevant features of IDPs are their promiscuous interactions and structural flexibility. The ability of IDPs/IDRs to interact with their multiple partners arises from their structural variations among their free and the target-bound states. We have named the hotspots found in multiple IDP interactions as protein segments (ProS), which are important for molecular recognition by their targets^14,15^. Another example of the IDP molecular function is the “fly-casting mechanism,” which accelerates associations with partner molecules^16–18^. When a highly flexible IDR interconnects two functional domains and one of them is spatially fixed, the other domain can sweep out within a sphere of a fixed radius. Notably, this fly-casting function is considered to show little sequence dependence.

Recently, we and other researchers have proposed that IDPs serve important physiological function as protectants from environmental stresses, such as freezing and desiccation^19–22^. For example, plant dehydrins (DHNs) are well-studied cryoprotective IDPs^23–25^. The DHNs harbor one or more genetically conserved sequence motifs in the form of Lys-rich (K-), Tyr-rich (Y-), and Ser-rich (S-) segments^20^. Several unstructured segments derived from DHNs prevent inactivation of the model reporter enzyme, lactate dehydrogenase (LDH), during repeated freeze/thaw treatments^21,26^. A “molecular shield” effect was the proposed mechanism underlying this DHN cryoprotection^21,27,28^.

Although this molecular shielding is closely related to macromolecular crowding, the two processes are distinct. Molecular shielding may decrease stochastic self-collisions between target enzyme molecules during the occasional high concentrations occurring in repeated freeze–thaw cycles^21^. Two research groups have reported that a weak but clear correlation between the cryoprotective activity of various DHN segments and their mutants versus their hydrodynamic radius (Rh), whereas their amino acid compositions or sequences were not relevant^21,26,28,29^. We succeeded in expanding the concept of the molecular shield by showing that it is independent of the amino acid sequence and only depends on the intrinsic disorder of the protein. We demonstrated that some fragments of genetically unrelated IDPs randomly selected from the human genome also showed substantial cryoprotective activity against LDH deactivation^19^. Thus, the sequence independence of the molecular shield hypothesis is at least partially proven.

In the present study, we focused on the molecular shield effect as a neglected biophysical property of IDPs, and we examined its relationship to inhibition of amyloid fibril formation using the β-amyloid peptide (Aβ(1–42)) as a model system. First, we attempted to distinguish between molecular shielding and macromolecular crowding. In general, the presence of macromolecular crowding agents accelerated protein precipitation, aggregation, and amyloid fibril formation. For example, the presence of 150 mg/mL PEG 600 and PEG 3000 accelerated the fibril formation of α-synuclein by approximately 50- to 100-fold^30^. The mechanism of this acceleration involves the exclusion volume effect, which packs the disordered and elongated α-synuclein monomer into the compactly folded, fibrillization-prone conformation. Similarly, another report has indicated that an intermediate range of concentrations of Ficoll and dextran could accelerate fibril formation of Aβ(1–40) from 0.75 to 12%^31^. By contrast, other reports have shown that crowding reagents can inhibit fibrillation of amyloid or other model proteins^32,33^. Extrapolating from the cryoprotection mechanism of IDPs, we hypothesized that the presence of IDPs at a relatively low concentration could inhibit Aβ(1–42) fibrillization.

## Results

### IDPs suppressed Aβ(1–42) amyloid fibril formation at lower concentration than that of molecular crowding polymers

**Figure 1:**
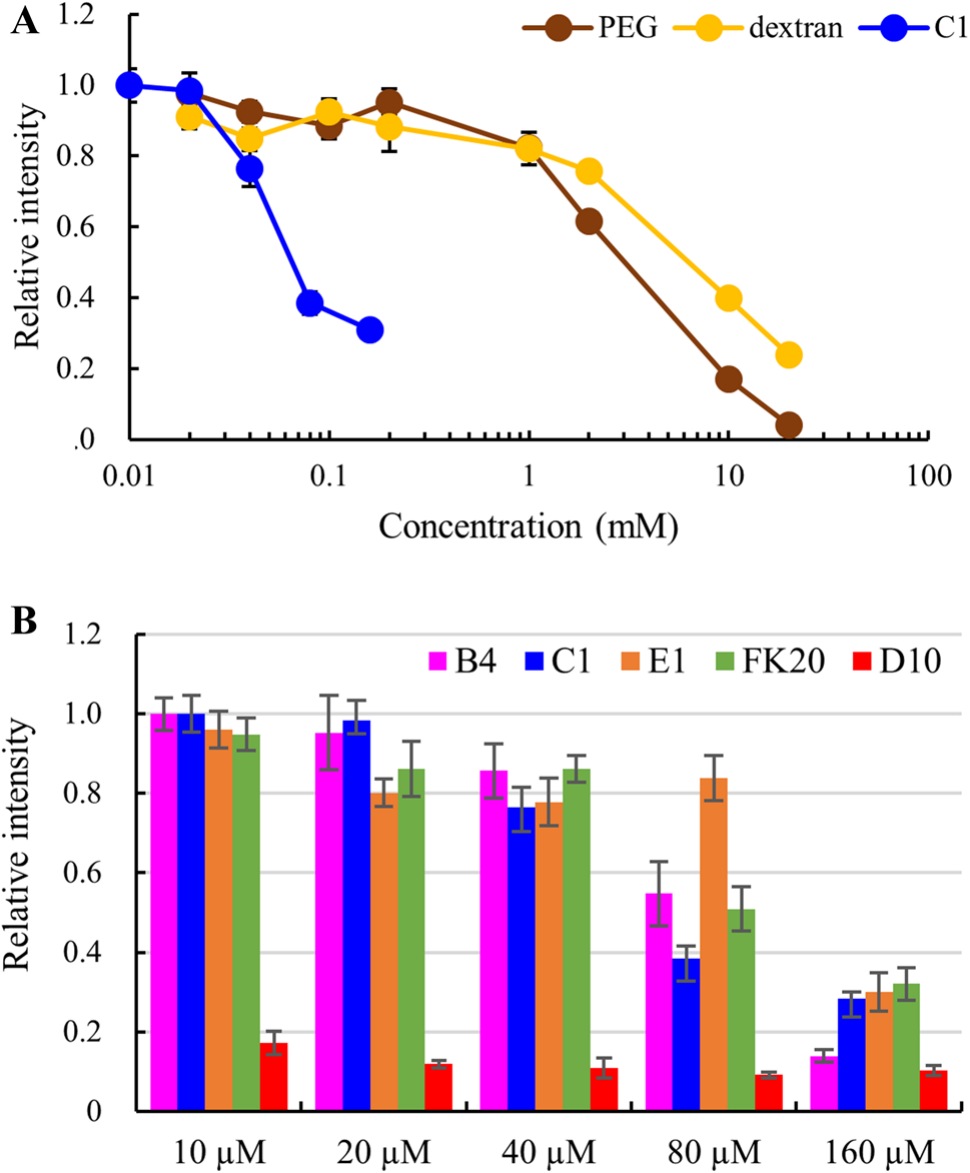
The effect of human genome–derived IDPs and other crowding agents on Aβ(1–42) amyloid fibril formation probed using thioflavin T (ThT). **(A)** Aβ(1–42) peptide (20 µM) was incubated for 72 h at 37 ℃ in the absence or presence of 0.01–20 mM IDP (C1) (blue), or PEG 6000 (brown), dextran 6 (yellow). The formation of Aβ(1–42) fibrils was monitored by ThT fluorescence and normalized to 1.0 with the intensity of the Aβ(1–42) alone. **(B)** 20 µM Aβ(1–42) peptide was incubated for 72 h at 37 ℃ in the presence of 10–160 µM IDPs. IDP-B4 (purple), C1 (blue), E1 (orange), FK20 (green), and D10 (red). Error bars, s.d. from three independent experiments.

Some IDPs, including plant LEA gene products and DHNs, are believed to suppress aggregation of other proteins by stochastic interference of direct contact of denatured proteins^20,21,27,28^. The mechanism is known as the “molecular shield effect.” Accordingly, we hypothesized that other IDPs can similarly suppress amyloid fibril formation. We examined the inhibitory activity of our selected human genome-derived IDPs against Aβ(1–42) as a model system (Table 1). Since the molecular shield effect is considered to function for IDPs and for other molecular crowding agents, we used PEG 6000 and Dextran 6 as positive controls representing synthetic and biological hydrophilic polymers, respectively. Figure 1A shows the inhibitory activity against fibrillization by one of the human genome-derived IDPs (IDP-C1, molecular weight 4,150) versus the activity of PEG 6000 and Dextran 6. IDP-C1 suppressed amyloid fibril formation at a dilute concentration (IC_50_ = 67.8 μM, see below). The presence of a large molecular excess of PEG 6000 or Dextran 6 (IC_50_ values of 4.1 mM and 7.7 mM, respectively) also suppressed Aβ(1–42) fibrillization. These concentrations correspond to approximately 200 and 400 molecular equivalence, indicating that IDP-C1 suppressed fibril formation at concentrations two orders of magnitude lower than that of the other crowding agents.

**Table 1:** Amino acid sequences and molecular weights of the human genome–derived intrinsically disordered protein (IDP) samples used in this study.

IDP name	RefSeq ID	Start	End	Length	Sequence	M.W
B4	NP_001317	1	44	44	MSGDGATEQAAEYVPEKVKKAEKKLEENPYDLDAWSILIREAQV	4,951
C1	NP_570859	1	36	36	MAALRYAGLDDTDSEDELPPGWEERTTKDGWVYYAK	4,150
D10	NP_002537	24	62	39	FPPKYLHYDEETSHQLLCDKCPPGTYLKQHCTAKWKTVC	4,610
E1	NP_005222	305	342	37	GFGGKYGVQKDRMDKNASTFEDVTQVSSAYQKTVPVE	4,068
FK20	NP_002537	24	43	20	FPPKYLHYDEETSHQLLCDK	2,464

**Figure 2:**
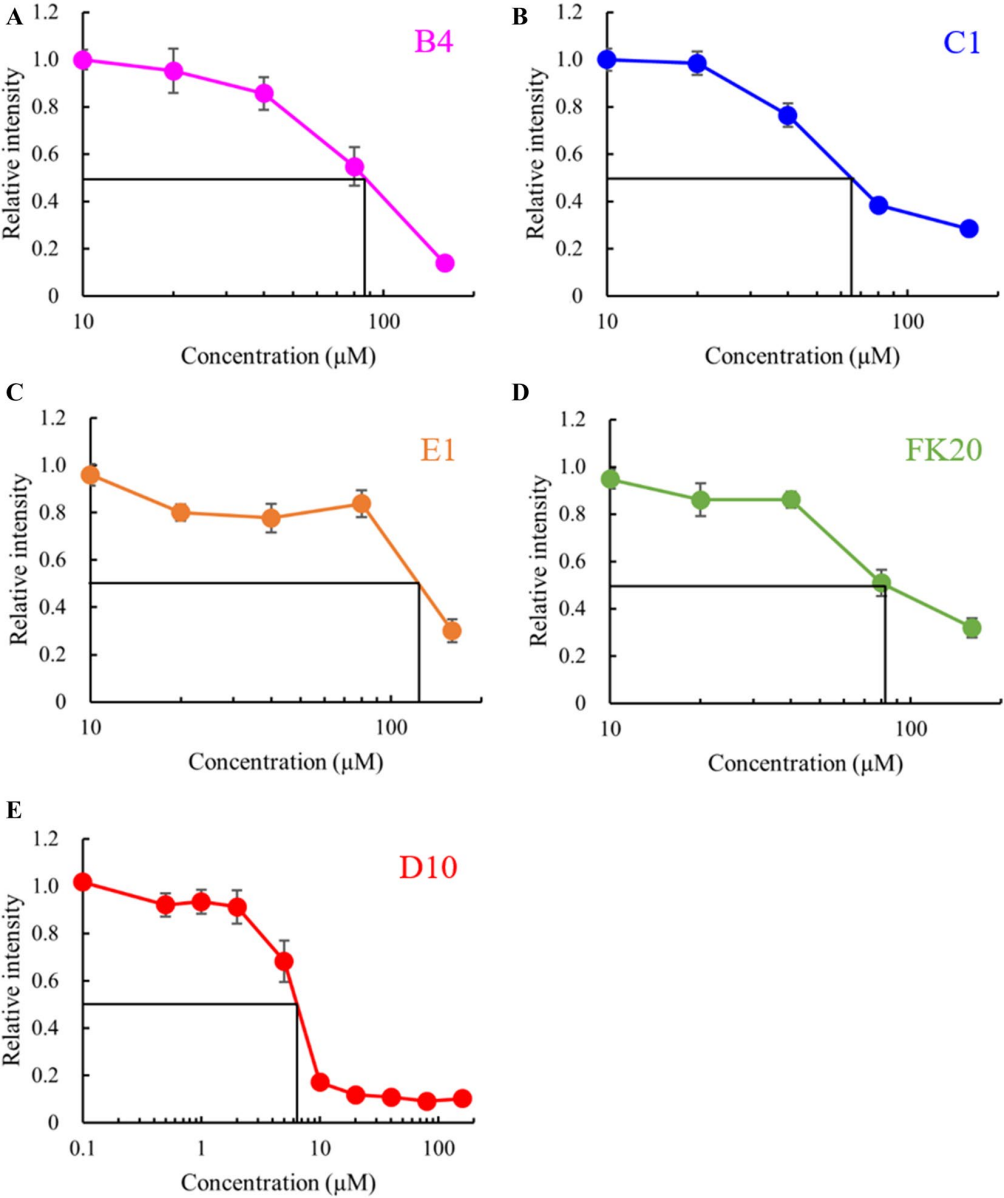
Estimation of apparent IC_50_ value of IDPs against Aβ(1–42) fibril formation. **(A)** B4, **(B)** C1, **(C)** E1, **(D)** FK20, **(E)** D10. Error bars, s.d. from three independent experiments.

We also examined whether the inhibitory effect of IDP-C1 against fibril formation was common to the other selected IDPs by testing their concentration dependence of fibril formation inhibition, calculating their IC_50_ values, and comparing them to IDP-C1 (Figs. 1B, 2). The IC_50_ values against 20 μM of Aβ(1–42) are summarized in Table 2. Three of the four tested IDPs (IDP-B4, C1, and E1) showed similar IC_50_ value ranges (67 to 131 μM) corresponding to 3 to 7 molecular equivalences of Aβ(1–42). This experiment revealed that IDP-D10 was approximately 10 times more potent at inhibiting fibril formation (IC_50_ value of 6.8 μM) when compared to the other three IDPs. This corresponded to one third of the molecular equivalence of the Aβ(1–42) monomer. By contrast, the FK20 peptide, which consists of the N-terminal 20 amino acids of IDP-D10, exhibited a weaker inhibition (IC_50_ value of 82 μM) and had an IC_50_ value similar to those of the other three IDPs. The order of the inhibition of fibril formation was therefore D10 > C1 > FK20 > B4 > E1.

**Table 2:** Comparison of inhibitory activity (IC_50_) of each IDP and crowding agent on Aβ(1–42) fibril formation.

IDP	IC_50_ (µM)
B4	90
C1	68
D10	6.8
E1	130
FK20	82
PEG6000	4,100
DEX6	7,700

This observation led us to consider that the mechanism of fibrillization inhibition differed between IDP-D10 and the other IDPs. IDP-D10 could possibly act against the Aβ(1–42) oligomer rather than Aβ(1–42) monomer, since IDP-D10 treatment inhibited fibrillization by 80% even at a half molecular ratio of 20 μM Aβ(1–42) (Fig. 2E). Although FK20 was still a potent amyloid inhibitor, with activity similar to the other IDPs, it was far less inhibitory when compared to IDP-D10. Thus, this high activity of D10 may require either the C-terminal half or the full length of the IDP-D10 sequence. Conversely, the weaker inhibitory activities (IC_50_ values ranging between 3 to 7 molecular equivalence) of the three IDPs and FK20 may be independent of the amino acid sequence, since all four IDPs (including IDP-D10) were unrelated to each other in terms of their amino acid sequences^19^. Therefore, the range of IC_50_ values for amyloid fibril inhibition reflected the molecular shield effects of the IDPs.

### Observation of Aβ(1–42) fibrils in the absence and presence of IDPs

**Figure 3:**
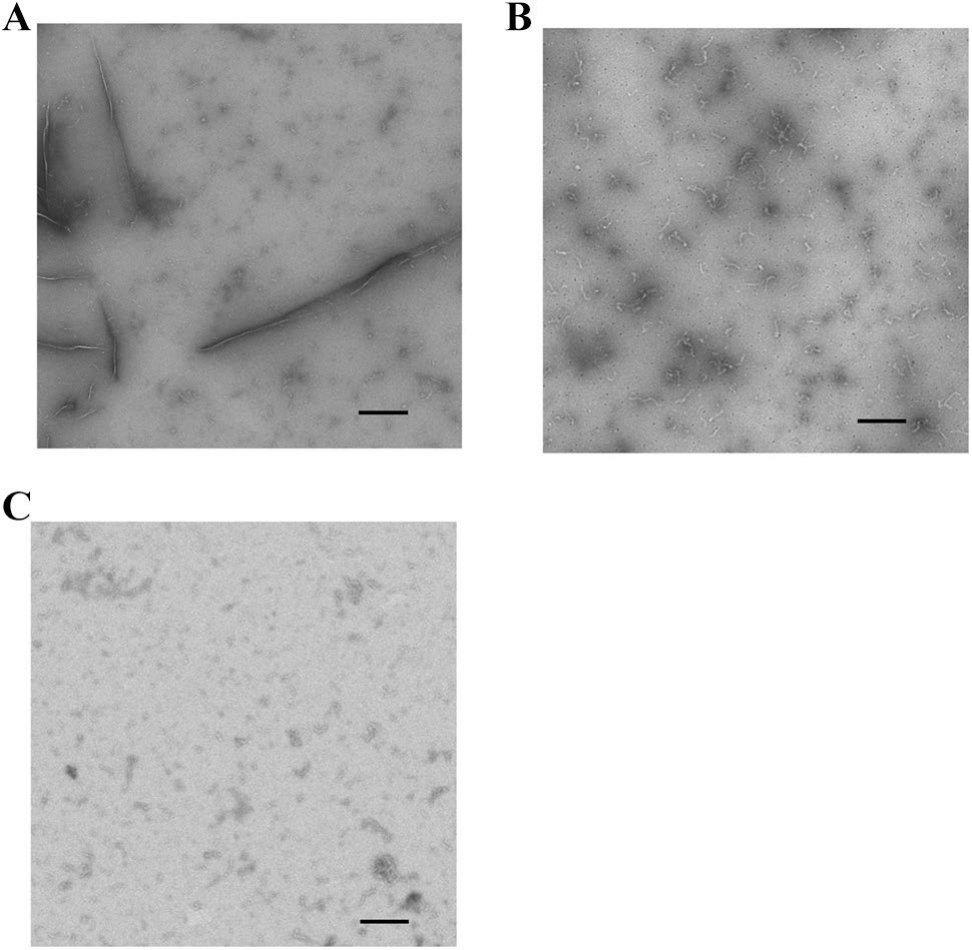
Observation of Aβ(1–42)-derived amyloid fibrils by negatively stained transmission electron microscopy with or without IDPs. TEM images of 10 µM Aβ(1–42) were obtained after 24 h of incubation either alone **(A)** or in the presence of 40 µM IDP-C1 **(B)** or 10 µM IDP-D10 **(C)**. Scale bars indicate 200 nm.

The thioflavin T (ThT) assay results suggested that the inhibitory effects on amyloid fibril formation by Aβ(1–42) might be independent of the IDP amino acid sequence. We investigated this further by examining how these IDPs interfered with amyloid formation by observing the shapes of partly inhibited Aβ(1–42) fibrils by negative staining electron microscopy (Fig. 3). In the absence of IDP, we observed several long straight fibrils (Fig. 3A). In the presence of 40 μM IDP-C1 and 10 μM IDP-D10, the long fibrils or amorphous aggregates were rarely observed; instead, we saw short fibrils and dots (Fig. 3B, C). Comparison of the fibrils formed with in the presence of IDP-C1 and IDP-D10 revealed fewer short fibrils in the presence of D10 than in the presence of C1. These results were consistent with the ThT assay findings that showed a more potent inhibition of fibrillization by D10 than by C1.

### Nuclear magnetic resonance (NMR) titration experiments showed the absence of a specific interaction between Aβ(1–42) and IDPs

**Figure 4:**
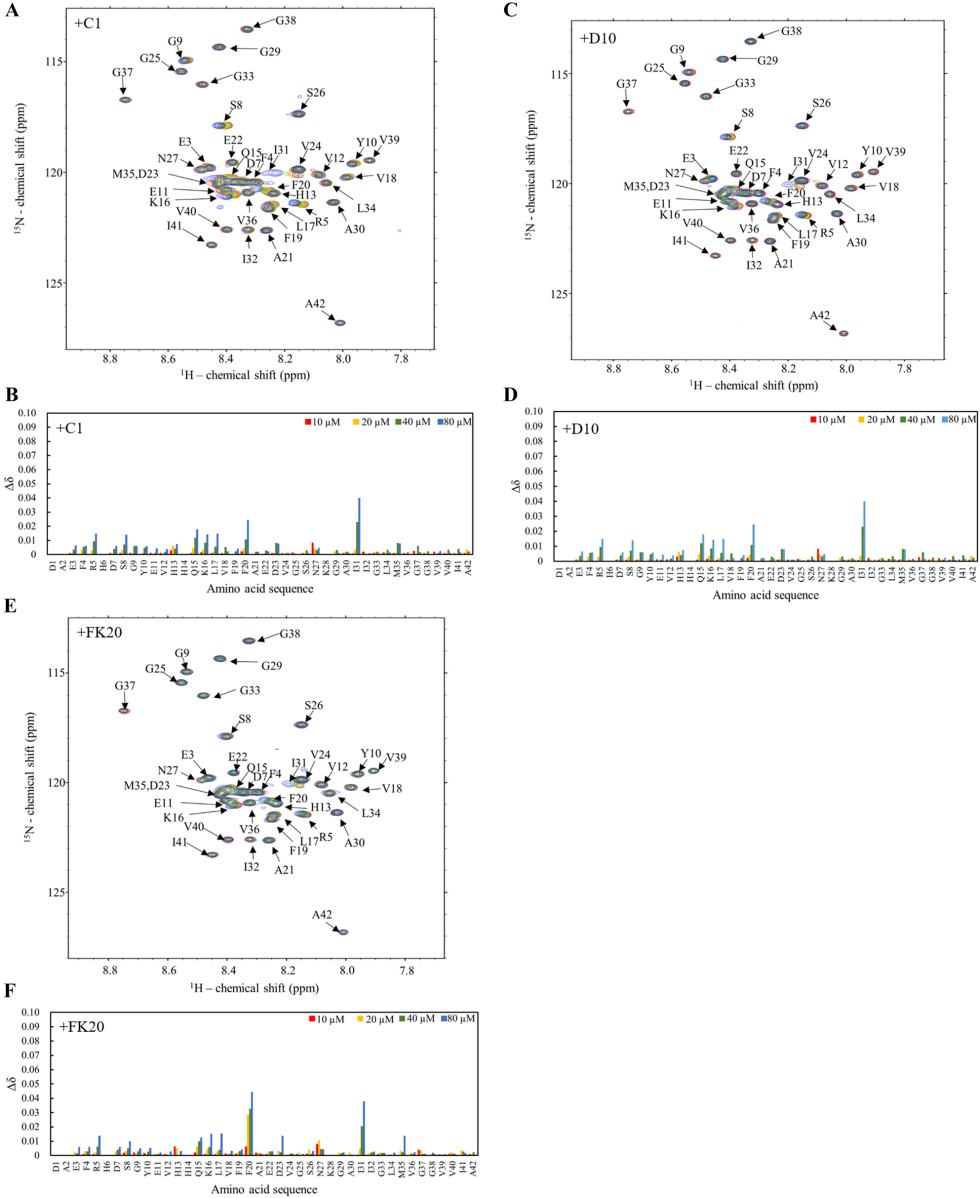
Absence of relevant interaction of IDPs with Aβ(1–42), assessed by solution nuclear magnetic resonance (NMR). **(A, C, E)** Overlay of the 2D ^1^H–^15^N HSQC spectra of Aβ(1–42) with various concentration of IDP-C1 at 15 ℃, pH 7.4. Aβ(1–42) (20 µM) with of 0 µM (cyan), 10 µM (red), 20 µM (yellow), 40 µM (green), and 80 µM (blue) of C1 **(A)**, D10 **(C)**, or FK20 **(E)**. **(B, D, F)** Normalized chemical shift perturbation Δδ derived from ^1^H–^15^N HSQC spectra of Aβ(1–42) with 80 µM C1 **(B)**, D10 **(D)**, or FK20 **(F)** were plotted against residue numbers of Aβ(1–42).

We also tested the possibility of a specific molecular interaction between Aβ(1–42) and IDPs using a chemical shift perturbation (CSP) experiment based on solution NMR, which can detect specific and non-specific ligand binding. We measured ^1^H–^15^N HSQC using 20 μM of ^15^N-labelled Aβ(1–42); this concentration was set to the same concentration of that of the ThT assays. As shown in Fig. 4, the NH signals showed a narrow signal dispersion typical of intrinsically disordered proteins and consistent with the spectra published in the literature. We concluded that the NMR samples of Aβ(1–42) were essentially in the monomer form before fibril formation; therefore, they were usable for further experiments.

Titration of the two IDPs (C1 and D10) and FK20 resulted in small but clear chemical shift changes in the NH signals (Fig. 4A–C, top panels). The magnitudes of the normalized chemical shift changes are plotted on the bottom panels of Fig. 4. More than 20 residues were affected by addition of the IDPs; however, the dispersion of the NH signals was still narrow, and likely remained intrinsically disordered. A Venn diagram of the top 20 residues that exhibited CSPs upon titration of IDPs and FK20 peptide is depicted in Figure S1. Overall, 18 residues were common to C1 and D10, suggesting the presence of a weak and non-specific interaction rather than a position-specific interaction between Aβ(1–42) and the IDPs. Comparison of the shifted residues of D10 (the most potent amyloid inhibitor) to FK20 (N-terminal half of D10) and C1 revealed that Val39 was the unique residue in D10. A specific interaction between the C-terminal half of D10 and Val39 of Aβ(1–42) is suggested as a key interaction for the high inhibitory activity of D10.

In the absence of IDPs, the NH signal intensities of Aβ(1–42) decreased after 60 h of NMR measurement without chemical shift changes (data not shown). Thus, although IDPs suppressed the amyloid fibril formation that is sensitive to ThT fluorescence, the formation of ThT-insensitive oligomers (or aggregates) was not ruled out.

### IDPs did not inhibit fibril formation in the presence of an excess amount of amyloid fibril seeds

**Figure 5:**
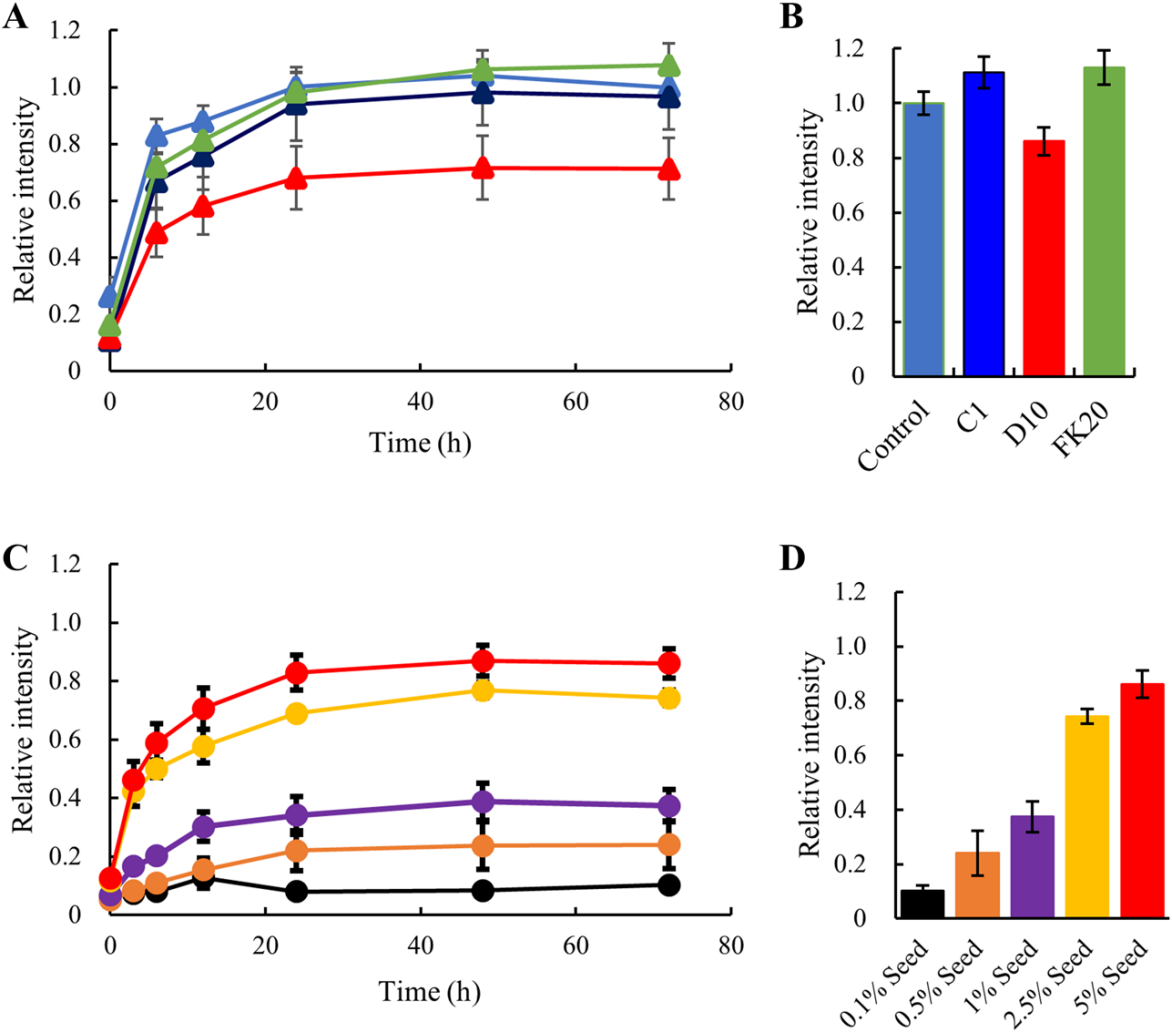
Reversal of IDP inhibition of fibril formation of Aβ(1–42) by addition of external amyloid seed. **(A)** Aβ(1–42) peptide (20 µM) was incubated for 72 h at 37 ℃ with 5% of external Aβ(1–42) amyloid seed in the presence of 80 µM C1 (blue), 80 µM FK20 (green), and 20 µM D10 (red). Thioflavin T (ThT) fluorescence intensity of the Aβ(1–42) alone with 5% seed was set to 1.0. **(B)** Comparison of relative ThT intensity from panel **(A)** at 72 h. **(C)** Aβ(1–42) (20 µM) peptide and 20 µM IDP-D10 was co-incubated for 72 h at 37 ℃ with various amounts of the external Aβ(1–42) amyloid seed. The seed amounts were 0.1% seed (black), 0.5% seed (orange), 1% seed (purple), 2.5% seed (yellow), and 5% seed (red), respectively. **(D)** Comparison of relative ThT intensity from panel **(C)** at 72 h. Error bars, s.d. from three independent experiments.

The process of amyloid fibril formation consists of two phases: the nucleation phase and the elongation phase^34^. In an ideal fibrillization experiment, these two phases appear as a lag-phase of ThT fluorescence enhancement followed by a rising phase, which correspond to the nucleation phase and the elongation phase, respectively. The fluorescence typically reached a plateau after several hours or days. In theory, the inhibition of amyloid fibrillization by the IDPs could act in both phases, even if the mechanism of action of the IDPs is via the molecular shield effect. We therefore tested whether IDPs still suppress fibril formation in the presence of the seed nuclei. The Aβ(1–42) amyloid seeds were prepared by breaking down the fibrils by sonication according to the literature, with a slight modification^35,36^. Examination of the inhibitory effect of the IDPs in the presence of 5% seeds (Fig. 5A, B) showed an elimination of the typical lag phase on the ThT assay profile and increases in both the elongation rate and the fluorescence intensity at the plateau.

Two reasons could explain the increase in the fluorescence intensity at the plateau phase. The first is that the increased amount of the amyloid seeds could have increased the total amount of fibrils. In the absence of fibrillization nuclei, the formation of the off-pathway oligomers or amorphous aggregates other than fibrils will compete with nucleation, thereby preventing fibril elongation and suppressing the total fibril amounts. In the presence of sufficient fibrillization nuclei, subsequent fibril elongation will be accelerated and may surpass the formation of off-pathway oligomers. The second possibility is that the highly homogenous amyloid seeds may promote the formation of more stable and longer fibrils than those fibrilized from intrinsic nuclei. This possibility would also explain the increase in the total amount of ThT-positive fibrils. In this study, we measured the IDP inhibition of amyloid formation at a sufficiently high concentration of the IDPs to inhibit amyloid formation (40 μM) even in the presence of amyloid nuclei. Therefore, the IDPs appear mainly to act on the nucleation phase rather than on the elongation phase.

We also determined the critical concentration of amyloid seeds that can induce fibrillization even in the presence of the inhibitory IDPs, by increasing amount of the seeds (Fig. 5C, D). An increase in ThT fluorescence was observed according to the increasing amount of the seeds in the presence of D10. At a seed level of 0.5%, which corresponds to 100 nM Aβ(1–42) monomer, no fibril formation occurred in the presence of 20 μM IDP-D10.

## Discussion

**Figure 6:**
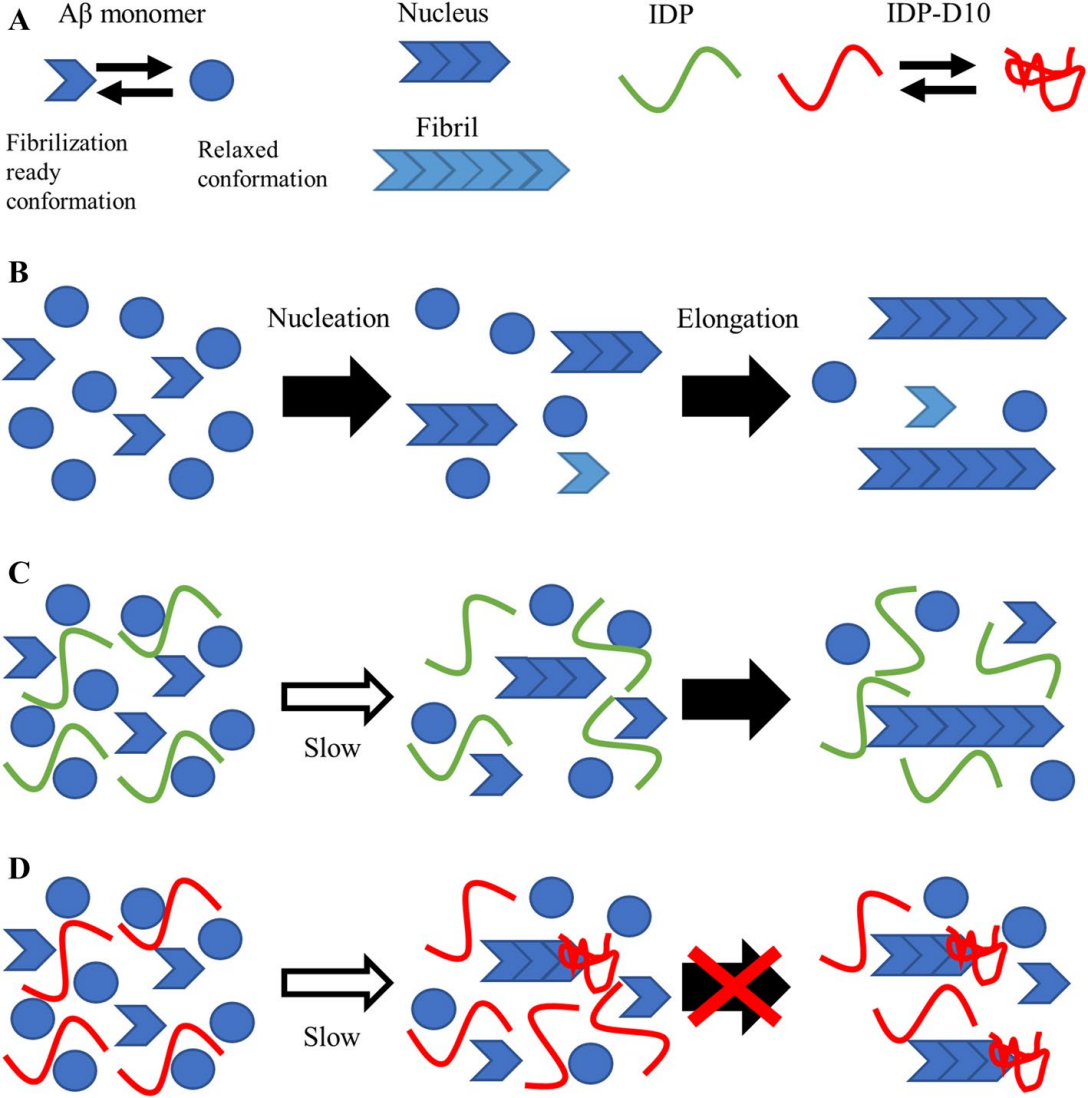
Schematic representation of molecular shield effect of human genome-derived IDPs against amyloid fibril formation of Aβ(1–42). **(A)** Aβ(1–42) monomer is considered to be an equilibrium between two conformations, the fibrillization-ready conformation (short arrow) and relaxed conformation (filled circle). Only when several fibrillization-ready Aβ(1–42) monomers encountered, the nucleus was formed (short pile of arrows). **(B)** The case of Aβ(1–42) alone. The nuclei are formed at the early nucleation phase, then the fibrils (long pile of arrow) grow by addition of fibrillization-ready monomers at the elongation phase. **(C)** The case of Aβ(1–42) and IDPs (except of D10). IDPs may not interfere equilibrium between fibrillization-ready and relaxed conformations of the monomer. IDPs may interfere the formation of the nucleus, thereby inhibiting fibril formation. **(D)** The case of Aβ(1–42) and IDP-D10. D10 probably binds to the nucleus and inhibits fibrillization.

We have demonstrated that IDPs suppressed Aβ(1–42) fibril formation through their molecular shield effect. We illustrated a possible scenario by which Aβ fibril formation was prevented by the molecular shielding effect (Fig. 6). In a solution solely containing Aβ(1–42) monomer, the rate-limiting step was the nucleation phase, in which several Aβ(1–42) monomers simultaneously interact to form the seed oligomer. Subsequently, the fibril starts to grow by continuous addition of Aβ monomers to the nucleus. We showed that the IDPs, except for IDP-D10, can interfere with the fibrillization at similar effective concentrations, and these inhibitory effects were overcome by addition of extra fibrillization nuclei. This suggested that the molecular shield is only effective during the nucleation phase and not during the elongation phase. In other words, the nucleation phase of Aβ fibrillization is more sensitive to impurities when compared with the elongation phase. It should be noted that all apparent IC50 values were apparently high compared to the other known Aβ fibrillization inhibitory peptides, such as the designed peptide V24P, that is artificial Aβ(1–40) variant with single amino acid substitution Val24-to-^D^Pro^37^. This peptide was reported to specifically form 1:1 complex with Aβ(1–42) and its IC50 is 30 μM. In turn, it is reasonable that the IC50 of fibrillization inhibition by molecular shield effect is rather high, since the effect was not due to the specific interaction, but probably by stochastic molecular contacts, that is the nature of the molecular shield phenomenon.

Many in vitro Aβ fibrillization studies, including the search for low molecular weight inhibitors, were designed to use a pure Aβ monomer solution. However, our results emphasize that the presence of other proteins, and especially IDPs from cerebrospinal fluid (CSF), the Golgi apparatus, or recycling endosomes, may have a substantial effect. The presence of only four molar excess (80 μM IDP versus 20 μM Aβ) was not markedly weaker than many reported IC_50_ values for small molecular amyloid inhibitors and the effect was amino acid sequence independent. In this study, we found that IDP-D10 had particularly potent amyloid inhibition activity. Since IDP-D10 showed an inhibitory effect on fibrillization at a lower concentration than that of Aβ, we assumed that D10 inhibited fibril formation by binding to the fibrillization nuclei or amyloid oligomers. This remarkably high inhibitory activity was only observed for D10 and not for FK20 peptide, which is the N-terminal half of D10, suggesting that the inhibitory activity is attributed to the C-terminal half of D10 or to the presence of both the N- and C-terminal halves of D10. Notably, the D10 did not have observable affinity for the Aβ monomer, as revealed by the NMR titration experiment. No fibrillization nuclei were observed in the NMR sample of ^15^N-labelled Aβ, probably because of their small population. The literature contains some reports of amyloid inhibitory peptides that act against amyloid oligomers. The examples include N-terminal disordered peptides derived from bovine prion protein precursor (bPrP), PrP(25–35) and PrP(108–119)^38^. However, we did not find any sequence similarity among IDP-D10, PrP(25–35), or PrP(108–119). Notably, IDP-D10 is a partial sequence of TNFRSF11B, which, unlike bPrP, is not expressed in the brain. Thus, TNFRSF11B is unlikely to act as either an amyloid inhibitor or an AD modulator in nature, although IDP-D10 showed a potent amyloid inhibition activity.

A healthy person’s cerebrospinal fluid (CSF) always contains soluble Aβ(1–42) monomer, and the concentrations of amyloid fibrils and aggregates are low. The amount of Aβ(1–42) in CSF gradually increases with age, and the CSF of familial Alzheimer's disease (AD) patients, who possesses genetic mutations in amyloid precursor protein (APP), contain higher concentrations of Aβ(1–42) when compared to healthy persons. Nevertheless, even with fibrillization-prone APP mutant genes, familial AD patients still do not develop AD at an early age^39^. The Aβ(1–42) concentration in CSF from normal, mildly cognitive impaired, and AD-developing individuals is one of the most sensitive clinical markers of AD diagnosis; however, the amount of Aβ(1–42) is higher in the CSF of normal individuals than in patients with AD^40^. The Aβ(1–42) concentration in the CSF starts to decrease and the concentration of oligomers may start to increase only when the symptoms of AD start to develop^41^.

This mystery could be explained by our observation that other cellular and extracellular proteins, including IDPs, may suppress amyloid nucleation, thereby preventing the progression of AD symptoms. Indeed, in the case of cerebral amyloid angiopathy, a disorder closely related to AD that is caused by fibrillated Aβ(1–40), exogenous seeding of the aggregates in rat brain accelerated the disease symptoms^42^. Several lines of evidence from exogenous inoculation of AD and other amyloidosis-related patient-derived brain homogenates suggested a self-propagation hypothesis of Aβ fibril formation^43–45^. Similarly, exogenous inoculation of the patient’s brain-derived Aβ(1–42) into AD-susceptible transgenic mice brain was shown to accelerate AD-like symptoms^46^. A striking structural variation in Aβ fibrils was shown to be associated with certain AD clinical subtypes^47^. All these examples lead to the hypothesis that amyloid fibril formation in living organisms is confined more strictly than biochemists previously supposed, mainly by the molecular shield effect of other existing proteins, including IDPs, against the nucleation phase of fibril formation.

## Conclusion

We demonstrated that four of five human genome-derived IDPs randomly selected and examined in this study inhibited fibril formation of Aβ(1–42). These inhibitory activities had IC_50_ values that ranged between 60 and 130 μM against 20 μM Aβ(1–42), and seemed to be independent of the IDP amino acid sequences. None of the IDPs exhibited specific binding to Aβ(1–42) monomer, as confirmed by the NMR experiments using ^15^N-labeled Aβ(1–42). Therefore, we concluded that the activity was truly a “molecular shield effect.” The IC_50_ values for anti-fibrillization were two orders of magnitude lower for the IDPs than for polyethylene-glycol and dextran. The IDP anti-fibrillization activities were abolished by adding external “seeds” for amyloid formation. Thus, the molecular shield effect of IDPs is more effective during the amyloid nucleation phase than during the elongation phase.

## Methods

### Reagents

PEG 6000, Dextran 6 from *Leuconostoc* spp. (molecular weight 6,000), and Thioflavin-T (ThT) were purchased from Sigma-Aldrich-Japan (Tokyo, Japan). The 0.5 × phosphate-buffered saline (PBS) used in this study was prepared by a 20-fold dilution of PBS (10 ×) solution purchased from Nacalai Tesque (Kyoto, Japan, cat. No. 27575–31).

### Preparation of Aβ(1–42) monomer

Monomeric and soluble Aβ(1–42) peptide was prepared as described previously^48^. In essence, Aβ(1–42) was expressed with N-terminal hexa-histidine and yeast ubiquitin (His × 6-Ub) as fusion tags. The ^15^N-labeled recombinant proteins used for NMR experiments were expressed in *Escherichia coli* BL21(*DE3*) cells in M9 minimal medium in the presence of ^15^NH_4_Cl as the sole nitrogen source. The Aβ(1–42) fusion protein was expressed as inclusion bodies, collected by centrifugation, and solubilized in denaturing buffer containing 6 M guanidine hydrochloride. The Aβ(1–42) fusion protein was purified using Ni Sepharose 6 Fast Flow (GE Healthcare UK) and refolded on the column by stepwise dilution of guanidine hydrochloride. After removal of the His × 6-Ub tag, the Aβ(1–42) was further purified by high-performance liquid chromatography (HPLC) using a ZORBAX 300Extend C18 column (Agilent Technologies, USA). The purified Aβ(1–42) was lyophilized twice, first in 30% acetonitrile containing 0.1% trifluoroacetic acid (TFA) and then in 1,1,1,3,3,3-hexafluoro-2-propanol (Kanto Chemical, Japan).

### Preparation of human-genome derived IDPs

The human-genome derived IDPs were selected and prepared as previously described^19^. In brief, bovine viral diarrhea virus N-terminal autoprotease, N(pro), was used as a fusion tag^49^. After removal of the tag, the IDPs were further purified by reversed phase HPLC (Cosmosil 5C_18_-AR-300, Nacalai Tesque, ϕ4.6 mm × 250 mm), lyophilized, and stored at – 30 ℃ until use.

### ThT fluorescence assay

The fibril formation of Aβ(1–42) and its inhibition by the IDPs and the polymers were monitored by fluorescence enhancement upon ThT binding to amyloid. The fluorescence was recorded with an EnSpire Multimode Plate Reader (PerkinElmer, Boston, MA USA) with excitation and emission wavelengths set at 440 and 484 nm, respectively. The lyophilized Aβ(1–42) was dissolved in dimethyl sulfoxide (DMSO), immediately diluted in 0.5 × PBS, and then added to the IDP solution (in 0.5 × PBS) containing 1 mM ThT. The samples were added to a 96 well plate (PerkinElmer) and incubated at 37℃ without rotation. The ThT and protein concentrations were 20 μM and 25 μM, respectively. The fluorescence intensity was measured from 0 to 72 h.

### Transmission electron microscopy

For negatively stained electron microscopy samples, 10 μM (25 μg/mL) Aβ(1–42) was allowed to form amyloid fibrils in 0.5 × PBS (pH 7.4) at 37 °C (310 K), with or without IDP-C1 or IDP-D10 (40 μM and 10 μM, respectively). The amyloid fibrils were allowed to form for 24 h without stirring. Aliquots (2 μL) of the amyloid fibrils were placed on a glow-discharged, carbon-supported copper grid (elastic carbon substrate on 400 Mesh Cu grids (Nisshin EM. Co., Ltd, Tokyo, Japan) and stained with 2 μL of 2% uranyl acetate. The samples were then washed once (2 μL) with the same uranyl acetate solution. Images were collected using a JEM-1010 electron microscope (JEOL, Co, Tokyo, Japan) operating at an acceleration voltage of 100 kV and recorded using a Tietz 4 K complementary metal oxide semiconductor TemCam-F416 camera (TVIPS GmbH, Gauting, Germany).

### NMR spectroscopy

NMR experiments were performed on a Bruker Avance III 900-MHz spectrometer equipped with a cryomagnetic probe. The lyophilized Aβ(1–42) was first dissolved in a small amount of *d*_*6*_*-*DMSO and then diluted with 0.5 × PBS (9:1 H_2_O-D_2_O, pH 7.4). The ^1^H–^15^N heteronuclear single quantum coherence (HSQC) spectra were recorded for 20 µM Aβ(1–42) dissolved in 96% 0.5 × PBS (9:1 H_2_O-D_2_O, pH 7.4) and 4% *d*_6_-DMSO (v/v) at 288 K. For NMR titration studies with IDP-C1, D10, and FK20, 64 scans of the transients of ^1^H–^15^N HSQC were accumulated for 192 TD1 points for Aβ(1–42) alone and in the presence of 0.5 eq, 1.0 eq, and 2.0 eq of IDPs. Because of reduced intensity of signals, 1,024 transients were accumulated in the presence of 4.0 eq IDPs. All the normalized chemical shift changes in the ^1^H–^15^N HSQC spectra upon ligand titration were calculated as Δδ_normalized_ = {Δδ(^1^H)^2^ + [Δδ(^15^N)/5]^2^}^1/2^, where Δδ(^1^H) and Δδ(^15^N) are chemical shift changes in amide proton and amide nitrogen, respectively.

### Preparation of Aβ(1–42) amyloid fibril seed

The amyloid fibril seed for the seeding experiment was prepared from the human Aβ(1–42) peptide according to the self-template protocol over the fourth generation^35,36^. The first-ancestor fibrils were grown by incubation of fresh monomer Aβ(1–42) solutions at 37 ℃ for periods of 48 h in an unstirred and unshaken tube The second generation fibrils were grown under the same condition by seeding fresh solutions with sonicated fragments (5% of total Aβ(1–42)) of the first generation fibrils. This process was repeated three times, and the fourth-generation fibrils were fragmented by sonication to prepare the fibrillization seeds.

## Supplementary information

Supplementary Information
