# Supplementary material for: Presence of intrinsically disordered proteins can inhibit the nucleation phase of amyloid fibril formation of Aβ(1–42) in amino acid sequence independent manner
Source: Sci Rep. 2020 Jul 23;10:12334. doi: 10.1038/s41598-020-69129-1 (PMC7378830; doi:10.1038/s41598-020-69129-1)
Supplement: Supplementary file 1 — Supplementary Information [file 41598_2020_69129_MOESM1_ESM.pdf]

Supplementary Information

Presence of intrinsically disordered proteins can inhibit the nucleation phase of amyloid fibril formation of Aβ(1–42) in amino acid sequence independent manner.

Koki Ikeda[1], Shota Suzuki[2], Yoshiki Shigemitsu[1,3], Takeshi Tenno[1,4], Natsuko Goda[1], Atsunori Oshima[2,5], and **Hidekazu Hiroaki**[1,4,5,*]

1 Laboratory of Structural and Molecular Pharmacology, Graduate School of Pharmaceutical Sciences, Nagoya University, Furocho, Chikusa-ku, Nagoya, Aichi, 464-8601, Japan

2 Laboratory of Structural Physiology, Graduate School of Pharmaceutical Sciences, Nagoya University, Furocho, Chikusa-ku, Nagoya, Aichi, 464-8601, Japan

3 School of Life Science and Technology, Tokyo Institute of Technology, Nagatsuda, 4259, Midori-ku, Yokohama, Kanagawa 226-8503, Japan

4 Cellular and structural physiology institute (CeSPI), Nagoya University, Furocho, Chikusa-ku, Nagoya, Aichi, 464-8601, Japan

5 BeCellBar LLC, Business Incubation Building, Nagoya University, Furocho, Chikusa-ku, Nagoya, Aichi, 464-8601, Japan

* Corresponding author: Hidekazu Hiroaki, e-mail: hiroaki.hidekazu@f.mbox.nagoya-u.ac.jp

Keywords

molecular shield effect, molecular crowding, intrinsically disordered proteins, solution NMR, Alzheimer's dementia, amyloid fibril inhibitor

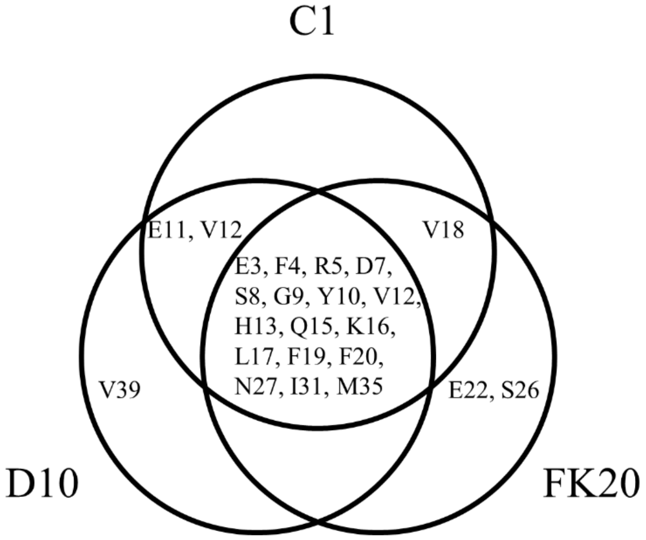

Supplementary Figure S1. Hot spots of chemical shift perturbation on Aβ(1–42) upon addition of IDPs.
Venn diagram plots of the top 15 residues of Aβ(1–42) that exhibited relatively large chemical shift changes upon addition of IDPs. Of the 15 residues with observable chemical shift change, the 13 residues were common upon the addition with the three independent IDPs.
